# Association Studies of HFE C282Y and H63D Variants with Oral Cancer Risk and Iron Homeostasis Among Whites and Blacks

**DOI:** 10.3390/cancers7040898

**Published:** 2015-12-09

**Authors:** Nathan R. Jones, Joseph H. Ashmore, Sang Y. Lee, John P. Richie, Philip Lazarus, Joshua E. Muscat

**Affiliations:** 1 Department of Pharmacology, Penn State University College of Medicine, Hershey, PA 17033, USA; nathan_jones8506@yahoo.com; 2 Department of Pharmaceutical Sciences, Washington State University, Spokane, WA 99202, USA; joe.ashmore@wsu.edu (J.H.A.); phil.lazarus@wsu.edu (P.L.); 3 Department of Neurosurgery, Penn State University College of Medicine, Hershey, PA 17033, USA; SYL3@psu.edu or slee1@hmc.psu.edu; 4 Department of Public Health Sciences, Penn State University College of Medicine, 500 University Drive, CH69, Hershey, PA 17033, USA; jrichie@hmc.psu.edu

**Keywords:** *HFE*, SNP, iron homeostasis, oral cancer, White, Black, genotyping

## Abstract

Background: Polymorphisms in the hemochromatosis (*HFE*) gene are associated with excessive iron absorption from the diet, and pro-oxidant effects of iron accumulation are thought to be a risk factor for several types of cancer. Methods: The C282Y (rs1800562) and H63D (rs1799945) polymorphisms were genotyped in 301 oral cancer cases and 437 controls and analyzed in relation to oral cancer risk, and serum iron biomarker levels from a subset of 130 subjects. Results: Individuals with the C282Y allele had lower total iron binding capacity (TIBC) (321.2 ± 37.2 µg/dL *vs.* 397.7 ± 89.0 µg/dL, *p* = 0.007) and higher percent transferrin saturation (22.0 ± 8.7 *vs.* 35.6 ± 22.9, *p* = 0.023) than wild type individuals. Iron and ferritin levels approached significantly higher levels for the C282Y allele (*p* = 0.0632 and *p* = 0.0588, respectively). Conclusions: Iron biomarker levels were elevated by the C282Y allele, but neither (rs1800562) nor (rs1799945) was associated with oral cancer risk in blacks and whites.

## 1. Introduction

The *HFE* gene produces a protein located on the surface of liver and intestinal cells that acts to detect iron levels. C282Y and H63D are the two most common missense polymorphisms in the *HFE* gene, and they both result in increased cellular iron uptake. The C282Y polymorphism prevents the protein from reaching the cell surface, resulting in an inability to accurately sense iron levels in the body [1,2,3]. Individuals homozygous for this allele can display severe iron accumulation and are at 20-fold increased risk for hepatocellular carcinoma (HCC) [4] and several large-scale studies have identified the C282Y polymorphism as a risk factor for HCC [5,6]. H63D leads to a reduced function protein [7], and H63D homozygotes and C282Y/H63D compound heterozygotes display mild iron accumulation [8,9,10]. Polymorphisms in the *HFE* gene are most common in individuals with northern European ancestry, but are also present in Blacks and Hispanics [11].

Hereditary hemochromatosis (HH) is one of the most common genetic disorders in persons of northern European descent that causes excessive iron absorption and storage. The primary cause of HH is the homozygous C282Y mutation (rs1800562) in the hemochromatosis (*HFE)* gene, which results in excess dietary iron absorption and deposition in various organs throughout the body. Heterozygotes have been reported to have higher mean transferrin saturation and ferritin levels than normal subjects, but rarely develop HH. The more prevalent H63D mutation (rs1799945) rarely causes HH. 

Iron deposition is toxic to the tissue in which it accumulates. Mechanisms for cellular injury include lipid peroxidation via iron-catalyzed free radical reactions, stimulation of collagen formation by activation of hepatic stellate cells, and interaction of reactive oxygen species and iron directly with DNA [12,13,14]. Altered iron metabolism affects carcinogenesis through a number of signaling pathways [15]. Because of the association between HH and HCC, and the proposed mechanisms by which iron deposition can lead to cellular injury and carcinogenesis, many studies have investigated the potential associations of *HFE* polymorphisms with various extrahepatic cancers, including colorectal cancer, hematological cancers, and estrogen-dependent cancers [16,17,18,19,20,21,22,23,24,25,26,27,28]. A meta-analysis of the association between *HFE* polymorphisms and 31 disease endpoints including cancers found associations of C282Y homozygotes with HCC only, while H63D was not associated with increased risk for any cancer examined [29]. However, there was insufficient power to draw conclusions for many other cancers. A more recent meta-analysis has linked the C282Y polymorphism with increased risk for colorectal cancer [30] and in a nested case-control study among participants in the European Prospective Investigation into Cancer and Nutrition (EPIC) study, the H63D polymorphism was associated with a significant increase in gastric cancer risk [31]. While altered iron status has been linked to head and neck cancer development [32], no studies on the possible impact of *HFE* polymorphisms have been reported to date. 

Iron deposition may play a role in the development of oropharyngeal cancer since oxidative stress has been implicated as a cause [33]. For example, iron deficiency was linked to cancers of the upper alimentary tract in Swedish women who presented with Plummer-Vinson syndrome [34], and mildly lower blood transferrin levels were associated with increased oral cancer risk in a subset of subjects from the current study who had blood samples obtained and measured for iron [32]. However, the lower blood iron levels among oral cancer cases was coupled with significantly elevated ferritin levels, indicating that these patients had higher intracellular iron stores. *HFE* variants are associated with higher serum ferritin concentrations [35]. Taken together with the fact that the *HFE* protein mediates intestinal iron uptake, we hypothesized that the C282Y and H63D polymorphisms are associated with increased oral cancer risk. 

To determine whether there is an increased risk of oral cancer in individuals with *HFE* mutations, we genotyped the C282Y and H63D polymorphisms in a case-control study of 301 oral cancer cases and 437 healthy controls. In a subset of patients from this population (*n* = 130), iron levels, ferritin levels, total iron binding capacity (TIBC), and transferrin saturation were stratified by *HFE* genotype status in order to determine the degree of iron accumulation in patients with *HFE* polymorphisms.

## 2. Materials and Methods

### 2.1. Study Population

Cases were patients diagnosed with primary squamous cell carcinoma of the oral cavity from 1994 to 1996 from three institutions [36]: All study subjects signed a consent form approved by the institutional review boards at Temple University Hospital (Philadelphia, PA, USA); Memorial Sloan-Kettering Cancer Center (New York, NY, USA); and New York Eye and Ear Infirmary (NYEEI, New York, NY, USA). Incident cases were defined as subjects diagnosed within 1 year prior to recruitment into the study. Greater than 90% of eligible cases were interviewed. Oral cancer was defined as squamous cell carcinoma of the oral cavity including gingiva (including hard palate and dorsal tongue), floor of the mouth, inner lip, soft palate, buccal mucosa, ventrolateral tongue, tonsil, oropharynx, and larynx. For all cases, confirmation of tumor type was pathologically confirmed. Controls (*n* = 437) were individuals without any prior diagnosis of cancer or present respiratory ailment. The controls consisted of friends, spouses, and spousal family members of cancer patients, or subjects receiving outpatient care for non-malignant related conditions. About 95% of eligible controls consented to participate in the study. Controls were frequency-matched to cases by age (±5 years), sex, race, month of diagnosis of the case subject, and institution. Two controls were sought for each case, although for most cases only one control was identified that met the criteria. 

Demographic information as well as information on tobacco use was collected for all cases and controls by administering a structured questionnaire by subject interview. Smoking levels were calculated as the sum of cigarettes, pipes, and cigars smoked according to the equivalents adopted previously, *i.e.,* 20 cigarettes = 4 cigars = 5 pipes = 1 pack [37], with our data calculated as pack-years (1 pack/day for 1 year = 1 pack-year). Never-smokers were defined as subjects who smoked < 1 pack-year. For many of the subjects that were interviewed, accurate lifetime alcohol consumption could not be estimated, but determinations were made on the peak amount of alcohol consumption. All cases who were drinkers drank for a minimum of 10 years. Alcohol consumption was classified as shots per day, where one shot = 12.9 g of 43% alcohol, which is roughly equivalent to 1 ounce of 86-proof hard liquor, one 3.6-ounce glass of wine, or one 12-ounce can of beer. Heavy drinkers consumed >7 shots per day, moderate drinkers 4–7 shots per day, and light drinkers 1–4 shots per day. Non-drinkers were subjects who consumed < 1 shot/day. Subjects with missing alcohol information are reported as “Missing” Clinical data pertaining to tumor site was collected from patient charts for all oral cancer subjects recruited into the study, and informed consent was obtained from all subjects.

### 2.2. Sample Collection and Processing

Buccal cell samples, isolated from cases at a follow-up examination, or normal oral tissue adjacent to the excised tumor isolated at the time of surgery from incident cases, were used for the analysis of polymorphic genotype frequencies in cases, whereas buccal cells were collected for the analysis in matched controls. Buccal cell samples were prepared as previously described [38]. Normal oral tissue obtained from cases during surgery was immediately frozen at −70 °C until snap-cooled in liquid nitrogen and homogenized in proteinase K-containing buffer. 

Fasting venous blood samples were obtained from 130 consecutively enrolled subjects at Memorial Sloan-Kettering Cancer Center. Pre-treatment samples were obtained from cases and controls from an antecubital vein into two 10 mL Trace Metal Vacutainer tubes containing heparin as the anticoagulant (Becton Dickinson, Franklin Lakes, NJ, USA) using a sterile 19-gauge needle. Blood was centrifuged to separate plasma, erythrocytes, and buffy coat. Erythrocytes were washed in saline three times. Plasma and erythrocyte samples were aliquoted for each assay, and all samples were immediately frozen and stored at −70 °C. 

### 2.3. Genotyping

*HFE* C282Y and H63D were genotyped using pre-designed Taqman 5’-exonuclease genotyping assays, according to manufacturer’s instructions. SDS 2.2.2 software was used for automated determination of genotypes (Applied Biosystems, Foster City, CA, USA). Briefly, allele-specific probes for rs1800562 and rs1799945 were labeled with the fluorescent dyes VIC and FAM. During extension, the 5′-exonuclease activity of the Taq polymerase separates the fluorophore from the non-fluorescent quencher. A post-amplification allelic discrimination run on the ABI 7900HT was used to determine genotype based on the relative amount of fluorescence of VIC and FAM. PCR reactions were carried out in a total reaction volume of 5 µL in 384-well plates using the ABI 7900 HT Sequence Detection System, and the thermal cycling conditions were 50 °C for 2 min, 95 °C for 10 min, and then 40 cycles at 95 °C for 15 s and at 60 °C for 90 s. Individuals involved in genotyping were blind to patient status. Quality control methods for PCR were previously described [39]. 

### 2.4. Serum Iron Analysis

Serum iron was measured spectrophotometrically as previously described [32,40]. Total iron binding capacity (TIBC) was determined by the method of Cook [41]. Percent transferrin saturation was calculated with the formula: serum iron/TIBC * 100, and serum ferritin was determined by a 2-site immunoradiometric assay [42].

### 2.5. Statistical Analysis

All statistical analyses were performed using SAS statistical software (version 9.2; SAS inst., Cary, NC, USA). Both the C282Y and H63D variants were found to be in Hardy-Weinberg equilibrium using χ^2^ goodness of fit tests. Odds ratios (OR) and 95% confidence intervals (CI) of association between cancer and SNP genotype were estimated using Fisher’s exact test. Potential confounding of the association between genotype and cancer risk by known risk factors was explored using Spearman rank correlation analyses and multivariate logistic regression models. Iron biomarker levels were compared between wild type individuals and individuals with the H63D and C282Y polymorphisms using an unpaired *t*-test.

## 3. Results

The basic demographic profile of the oral cancer case and control subjects is shown in Table 1. There were 201 White and 100 Black cases; 29% of White cases and 27% of Black cases were women. Among Whites, the mean ages of cases and controls were 60 ± 13 y and 59 ± 13 y, respectively (*p* = 0.22). In Blacks, the mean age of cases was 58 ± 10 y and the mean age of controls was 58 ± 13 y (*p* = 0.95). The mean pack-years of smoking for White and Black oral cancer cases (41.8 and 43.5 pack-years, respectively) was significantly (*p* < 0.01) higher than the mean pack-years observed for White and Black controls (17.6 and 18.4 pack-years, respectively). A significantly higher percentage (*p* < 0.01) of White cases were heavy drinkers (36.0%) compared to White controls (11.7%). Similarly, heavy alcohol consumption was significantly more prevalent (*p* < 0.01) in Black cases (49.5%) compared to Black controls (15.8%).

**Table 1:** Age, gender, smoking status, and alcohol consumption of 301 oral cancer cases and 437 controls.

	Oral Cases n (%)	Controls n (%)	*p*-value
*Whites*			
Mean Age *	60.1 ± 12.8	58.6 ± 13.3	0.22
Women	59 (29.4)	102 (33.4)	0.31
Pack-years *	41.8 ± 33.6	17.6 ± 29.0	<0.01
Alcohol consumption			<0.01
Heavy drinkers	58 (36.0)	31 (11.7)	
Moderate drinkers	32 (19.9)	34 (12.9)	
Light drinkers	21 (13.0)	68 (25.8)	
Non-drinkers	50 (31.0)	131 (49.6)	
Missing	40	37	
*Blacks*			
Mean Age *	58.3 ± 10.4	58.3 ± 13.3	0.95
Women	27 (27.0)	49 (36.6)	0.12
Pack-years *	43.5 ± 36.2	18.4 ± 30.1	<0.01
Alcohol			<0.01
Heavy drinkers	45 (49.5)	23 (15.8)	
Moderate drinkers	20 (22.0)	15 (10.2)	
Light drinkers	11 (12.1)	23 (15.8)	
Non-drinkers	15 (16.5)	85 (58.2)	
Missing	9	12	

* Mean ± standard deviation for continuous variables.

Both *HFE* SNPs were consistent with Hardy-Weinberg equilibrium. Among White controls, the minor allele frequencies (MAFs) of the *HFE* SNPs were 0.12 (H63D; rs1799945) and 0.03 (C282Y; rs1800562), which is similar to that in HapMap for individuals with European ancestry (0.15 and 0.06, respectively). The MAF in the Black controls was 0.03 for rs1799945, which was also similar to HapMap African ancestry from the Southwestern USA (0.02), and 0.02 for rs1800562; this SNP has not been previously reported for Blacks in HapMap.

As the number of individuals homozygous for either of the H63D (3 cases and 2 controls) or C282Y (1 case and 1 control) polymorphisms was low, risk analysis was performed by examining homozygous wild-type subjects *versus* subjects with at least one variant allele. No association was found between the H63D (rs1799945) and C282Y (rs1800562) SNPs and oral cancer risk with (Table 2) or without (results not shown) adjusting for covariates including gender, pack-years, and alcohol consumption. In race-specific analysis, there was also no association observed with either SNP. When stratified by sex, there was also no association between *HFE* genotypes and oral cancer. 

**Table 2:** Distribution of *HFE* SNPs and oral cancer risk.

	Cases, n (%)	Controls, n (%)	OR (95% CI) *
*Whites*			
rs1799945 (C>G), H63D			
CC	144 (82.8)	214 (79.6)	1.00 (reference)
CG + GG	30 (17.2)	55 (20.4)	0.81 (0.49–1.33)
rs1800562 (G>A), C282Y			
GG	188 (94.0)	280 (94.6)	1.00 (reference)
GA + AA	12 (6.0)	16 (5.4)	1.12 (0.52–2.41)
*Blacks*			
rs1799945 (C>G), H63D			
CC	86 (91.5)	116 (94.3)	1.00 (reference)
CG	8 (8.5)	7 (5.7)	1.54 (0.54–4.41)
rs1800562 (G>A), C282Y			
GG	98 (98.0)	129 (96.3)	1.00 (reference)
GA	2 (2.0)	5 (3.7)	0.53 (0.10–2.77)

* Estimated by logistic regression, adjusting for gender, alcohol, and pack-years.

Compound heterozygosity for the two SNPs was explored, and again no association with oral cancer risk was found for any combination of C282Y and H63D genotypes (results not shown). Because HPV infection is a major risk factor for cancers of the oropharynx, the data was stratified by tumor site excluding cases that might be related to HPV infection (results not shown). With the oropharyngeal cases excluded, no association was observed between either of the *HFE* polymorphisms and cancers risk.

**Table 3:** Association of *HFE* SNPs with biomarkers of iron metabolism.*

	rs1800562 (G>A), C282Y		
Biomarker	GG	GA + AA	*p*-value
Iron (µg/dL)	83.7 ± 28.2	109.4 ± 63.3	0.063
TIBC (µg/dL)	397 ± 89.0	321 ± 37.2	0.007
Ferritin (ng/mL)	166 ± 165	383 ± 248	0.058
Transferrin (%)	22.0 ± 8.7	35.6 ± 22.9	0.023
	rs1799945 (C>G), H63D		
Biomarker	CC	CG + GG	*p*-value
Iron (µug/dL)	84.0 ± 32.7	92.1 ± 21.4	0.357
TIBC (µg/dL)	396 ± 89.6	390 ± 108	0.820
Ferritin (ng/mL)	169 ± 170	183 ± 164	0.765
Transferrin (%)	22.3 ± 10.3	25.6 ± 9.7	0.251

* Values are mean ± SD.

The effect of H63D and C282Y on iron biomarker levels in 130 primarily white subjects is shown in Table 3. Individuals with at least one *HFE* C282Y allele exhibited significantly lower total iron binding capacity (*p* = 0.007) and a higher percent transferrin saturation (*p* = 0.023) than wild type individuals. Iron and ferritin levels approached significantly higher levels for subjects with at least one *HFE*^282Y^ allele (*p* = 0.063 and *p* = 0.058, respectively). For the H63D SNP, iron levels were not significantly different between wild type individuals and individuals with one or more copies of the polymorphic allele. Levels of TIBC, ferritin, and transferrin did not differ significantly by H63D genotype.

## 4. Discussion

The annual incidence of oral and pharyngeal cancer is approximately 75,000 cases in the United States [43], with Blacks having an increased prevalence of oral cancer compared to Whites [44,45]. The major risk factors for oral cancer include smoking, chronic alcohol intake, and low fruit consumption [46,47,48,49,50,51]. A 2010 study by Wu *et al.* found significant associations between SNPs in oxidative stress-related genes and cancers of the oral cavity [52], but little is known about the pro-oxidant effects of intracellular iron accumulation and its relation to oral cancer. Iron homeostasis is tightly regulated by duodenal absorption and distribution throughout the body since there are not regulated processes for iron excretion. The HFE protein controls iron absorption by regulating the production of the hepcidin hormone in the liver. After dietary iron is transported across the enterocyte apical membrane by divalent metal transporter 1 (DMT-1), it can be transported across the basolateral membrane by ferroportin [53]. Hepcidin acts as a negative regulator of iron absorption by binding to ferroportin and causing internalization followed by proteolysis, ultimately leading to decreased uptake from intestinal cells [53]. Hepcidin also regulates how much iron is distributed throughout the body by regulating ferroportin-mediated efflux from macrophages, which act as the body’s primary iron stores for whole body distribution [54]. HFE causes an increase in hepcidin levels by sensing when iron levels in the body are high, so a non-functional or reduced function variant of the *HFE* gene can lead to excessive absorption and an imbalance in iron homeostasis.

There is little data on *HFE* and oral cancer risk. A study that followed 36 patients with idiopathic hereditary hemochromatosis and documented extrahepatic cancers found that one out of 36 patients (~2.7%) developed oral cancer, which is much higher than the oral cancer prevalence in the general population (~0.1%) [55]. This result suggested that oral cancer might be more frequent in patients with iron overload, but it may also be due to chance or confounded by other factors (e.g., smoking). The current study examined the association between hemochromatosis genotypes and oral cancer risk, and the results indicate that *HFE* polymorphisms are not a risk factor for oral cancer in Whites and Blacks. The lack of a significant association seen for the C282Y allele, and especially in Blacks may be due to low statistical power, since the prevalence of this SNP was low. Because clinical penetrance of *HFE* polymorphisms is known to be higher in males [56,57], cases were also stratified by sex to account for the possibility that males and females have different susceptibilities to iron-induced carcinogenesis. HPV-related oropharyngeal cancer can be viewed as a different disease from other cancers of the oral cavity. The prevalence of HPV-associated oropharyngeal cancer has increased rapidly in the last few years, and was relatively less common during the enrollment period of this study. Because of this, the HPV status of the cases in this study is unknown. According to a 2010 article, the rate of HPV infection in oropharyngeal biopsies was around 40% in the U.S. in the 1990s, compared with 60-80% today [51]. In this study, 42% of the cases were found to be of the oropharynx (tonsil, palatine tonsil, lingual tonsil, base of tongue, tongue NOS, soft palate, uvula, palate NOS, pharynx NOS, and oropharynx). Stratification of the data by tumor site did not impact the association with the *HFE* C282Y and H63D genetic variants. These findings are consistent with the fact that genetic variants that cause imbalances in iron homeostasis are independent of other risk factors (e.g., smoking, HPV), so the findings should not be biased based on whether OPC was smoking-related or HPV-related.

One explanation for the lack of association between *HFE* genotypes and oral cancer cases may be because of the way excess iron is distributed in different tissues throughout the body. Although absorbed iron is distributed to all tissues of the body for vital cellular processes, excess iron is deposited differentially in the following tissues (decreasing order of severity): liver, pancreas, myocardium, pituitary gland, adrenal gland, thyroid and parathyroid glands, joints, and skin [58]. Iron is transported in the blood bound to transferrin, and uptake is mediated by transferrin receptors which endocytose the iron bound to transferrin [53]. The lack of association between *HFE* polymorphisms and oral cancer may indicate that there is not enough iron deposition in the relevant tissues to cause the cellular injuries that lead to neoplasia. Therefore, the functional characteristics of transferrin receptors and how they are regulated may lend important insights into understanding which types of cancers can result from the pro-oxidant effects of iron accumulation.

Another explanation for the lack of association with oral cancer risk may be due to the low clinical penetrance of the C282Y and H63D polymorphisms [56,57]. Even though TIBC, iron, ferritin, and transferrin saturation were all significantly or nearly significantly different in individuals with one or more copies of the C282Y polymorphism, the frequency of clinically elevated levels was not different between wild type and polymorphic individuals (data not shown). Normal iron biomarker levels were considered to fall within the following ranges [32]: iron, 60–170 µg/dL; ferritin 12–300 ng/dL; TIBC 240-450 µg/dL; transferrin saturation 15-45%. Because the pro-oxidant effects of excess iron deposition would be more likely to occur in individuals with clinically elevated iron levels, this study may underestimate the role of iron in oral cancer risk. Future studies examining individuals with *HFE* gene polymorphisms and clinically elevated iron levels should be conducted in order to better estimate how HH and iron levels may affect oral cancer risk. Because iron accumulation has only been reported in C282Y and H63D homozygotes and compound heterozygotes, larger case-control studies may be necessary to detect an association between these rarer genotypes and cancers of the oral cavity. In addition, because there are other genes that affect iron levels, it may be necessary to study the interaction of polymorphisms in multiple genes involved in iron regulation. A study by Wu *et al.* found this to be true for genes involved in oxidative stress; no single polymorphism affected oral cancer risk, but combinations of polymorphisms in the manganese superoxide dismutase, myeloperoxidase, catalase, and glutathione peroxidase 1 genes were associated with significantly increased risk of oral cancer [52].

In previous studies [32], significantly elevated ferritin levels and significantly reduced levels of the potent antioxidant glutathione were observed among oral cancer cases. This indicated that the oral cancer cases were under a higher oxidative stress burden, to which imbalances in iron homeostasis were hypothesized as a contributing factor. A previous study found that genetic factors accounted for a substantial portion of the variance in iron biomarkers (23%–66%), but that the C282Y and H63D *HFE* polymorphisms accounted for <5% of the overall variance that was observed [59]. In the same study, serum ferritin levels differed less than the other biomarkers between individuals with and without *HFE* polymorphisms, which agrees well with the findings in the current study that *HFE* polymorphisms were not associated with the significantly higher ferritin levels observed in oral cancer cases. It is well known that ferritin levels can increase in response to many factors including chronic disease [60], even when blood iron levels indicate an iron deficiency [61]. Taken together, these findings suggest that it may be necessary to implement a more comprehensive genetic approach involving numerous genes involved in iron homeostasis in future studies in order to investigate the potential link between iron levels and oral cancer risk.

## 5. Conclusions 

These studies demonstrate that a single nucleotide polymorphism in the hemochromatosis (*HFE*) gene affects iron metabolism. *HFE* polymorphisms were unrelated to head and neck cancer risk. 

## Abbreviations

HH, hereditary hemochromatosis; *HFE*, hemochromatosis; SNP, single nucleotide polymorphism; MAF, minor allele frequency; TIBC, total iron binding capacity; HCC, hepatocellular carcinoma.
